# Subjective Cognitive Decline and Frailty Trajectories and Influencing Factors in Japanese Community-Dwelling Older Adults: A Longitudinal Study

**DOI:** 10.3390/jcm12185803

**Published:** 2023-09-06

**Authors:** Seongryu Bae, Hiroyuki Shimada, Sangyoon Lee, Keitaro Makino, Ippei Chiba, Osamu Katayama, Kenji Harada, Hyuntae Park, Kenji Toba

**Affiliations:** 1 Department of Health Care and Science, Dong-A University, 37 Nakdong-daero 550, Saha-gu, Busan 49315, Republic of Korea; htpark@dau.ac.kr; 2 Department of Preventive Gerontology, Center for Gerontology and Social Science, National Center for Geriatrics and Gerontology, 7-430 Morioka, Obu 474-8511, Aichi, Japan; shimada@ncgg.go.jp (H.S.);; 3 Tohoku Medical Megabank Organization (ToMMo), Tohoku University, 2-1 Seiryo-machi, Aoba-ku, Sendai 980-8573, Miyagi, Japan; ippei.chiba.a3@tohoku.ac.jp; 4 Tokyo Metropolitan Hospital and Institute of Gerontology, 35-2 Sakae-cho, Itabashi-ku, Tokyo 173-0015, Japan

**Keywords:** subjective cognitive decline, frailty, joint trajectory, older adults, age-related changes

## Abstract

We studied frailty and subjective cognitive decline (SCD) trajectories in older Japanese adults and evaluated the influence of various factors on these trajectories. We analyzed data from 1157 non-demented adults aged 70 and above from 2013 to 2019. Frailty was assessed using the self-administered Kihon Checklist (KCL), a Japanese frailty index. SCD was evaluated using the questionnaire of the Subjective Memory Complaints scale. Through group-based joint trajectory models, we discerned three frailty trajectories: non-progressive (*n* = 775), moderate progressive (*n* = 312), and rapid progressive (*n* = 70); and three SCD trajectories: non-progressive (*n* = 302), moderate progressive (*n* = 625), and rapid progressive (*n* = 230). Individuals in the rapid progressive SCD trajectory had a 32.2% probability of also being in the rapid progressive frailty trajectory. In contrast, those in the non-progressive SCD trajectory had zero probability of being in the rapid progressive frailty trajectory. Both the rapid progressive frailty and SCD groups combined had a higher incidence of depressive symptoms and slow gait speed. Our results have found that frailty and SCD share a similar trajectory in Japanese older adults. Additionally, rapid progressive frailty and SCD were associated with the highest risk of depressive symptoms and slow gait speed. Thus, interventions targeting both frailty and cognitive decline should prioritize mental health enhancement and gait speed improvement.

## 1. Introduction

Japan’s population is expected to have the highest proportion of older adults in the world by 2050, with 39.9% of the national population aged 65 years or older [1]. Concerns associated with the quality of life also increase with an increasing aging population. Dementia is one of the major causes of poor quality of life in older adults [2]. Therefore, it is critical to identify modifiable risk factors for dementia to develop early and novel prevention strategies. Frailty and subjective cognitive decline (SCD) are risk factors for dementia. Subjective cognitive complaints are frequently observed in the older adult population. These self-evaluations of impairment in cognitive functions, with unimpaired performance on cognitive tests, indicate the presence of SCD [3]. Longitudinal studies have shown that individuals who report subjective memory complaints are likely to experience twice as much cognitive decline in the future and have a higher incidence of dementia than those who do not [4]. A meta-analysis study reported that approximately 2.3% and 6.6% of older adults with SCD would progress to mild cognitive impairment (MCI) and dementia per year, respectively. Over a five-year period, 24.4% of those with SCD will develop MCI, while 10.9% will progress to dementia, compared with 4.6% of those without SCD complaints [5]. The predictive value of SCD appears to be in the early stages of the neuropathologic process, and the presence of SCD has been associated with poorer mental health and lower quality of life [6]. 

Frailty is a state of vulnerability to poor resolution of homeostasis when exposed to a stressor because of age-related cumulative deficits across multiple physiological systems [7]. Frailty is also associated with cognitive decline, dementia, and pathology of Alzheimer’s disease (AD) [8]. Although many scales have been developed for the diagnosis of frailty, there is still no standard recommended method for diagnosis because of the condition’s multifactorial etiopathogenesis. Additionally, because frailty is clinically considered a pre-disability from physical, social, or psychological aspects, older adults’ functioning should be assessed across multiple domains to identify frailty. The Kihon Checklist (KCL) is a self-reported comprehensive questionnaire comprising 25 questions covering multiple domains of instrumental activities of daily living, physical function, oral function, nutrition, cognition, social activity, and depressive mood. It has been validated as a screening tool for frailty and was shown to have good to excellent accuracy [9]. Previous studies have shown that KCL is useful in predicting incidence of long-term care insurance (LTCI) certification [9] and dementia [10]. Thus, the KCL seems to be an efficient screening tool for identifying frailty in the primary care setting or in outpatient clinics to promote public health. 

The association between frailty and SCD, both independent risk factors for dementia appearing early in the course of the disease, has not been investigated. One prior cross-sectional study showed that the frailty component is related to SCD before the presence of overt dementia, suggesting that this association is present before overt cognitive impairment [11]. However, information on the evolution of the natural history of frailty and cognition over time is scarce. Additionally, it is unclear whether and to what extent the joint trajectories of frailty and SCD impact health outcomes among older adults. From the perspective of primary risk prevention, given the nature of the transition of frailty and cognitive function over time, it is important to investigate the differences in frailty and SCD over time among individuals. Therefore, the purpose of this study was to identify joint trajectories of frailty and SCD and investigate the related factors of the identified heterogeneous classes of frailty and SCD trajectories among community-dwelling Japanese older adults. 

Based on the above, we hypothesized that trajectory groups within the participants would exhibit different rates of change in frailty and SCD over time and would overlap in membership between frailty and SCD trajectory groups. Additionally, we hypothesized that there would be differences in main characteristics between the frailty and SCD trajectory groups.

## 2. Materials and Methods

### 2.1. Participants

The participants were recruited from a sub-cohort of the National Center for Geriatrics and Gerontology Study of Geriatric Syndromes (NCGG-SGS) [12], conducted in 2013 in the Midori Ward of Nagoya city, Aichi Prefecture, Japan. Of the 5257 individuals, 2145 were selected as participants after applying the following inclusion criteria from the ORANGE registry (Organized Registration for the Assessment of dementia on Nation-wide General consortium toward Effective treatment in Japan): (1) normal general cognitive functioning (≥24/30 Mini-Mental State Examination scores); (2) normal objective cognitive functioning (excluded people with MCI as indicated by an age- and education-adjusted score at least 1.5 standard deviations below the reference threshold in one or more specific cognitive domains, including memory, attention, executive function, and processing speed, all of which are commonly used for detailed neuropsychological assessment); (3) not having severe health problems including dementia, stroke, depression, and Parkinson’s disease; (4) no evidence of functional dependency (such as supervision or external assistance in performing activities of daily living); (5) no long-term care needs or support; and (6) not being enrolled in other studies. ORANGE registry documents were sent to the 2145 participants, and consent was obtained from 1157 participants. Since the baseline assessments in 2013, all participants were invited to participate in annual assessments from 2017 through 2019. The total number of observations was 6900 during the follow-up period. The study protocol was developed in accordance with the Declaration of Helsinki and was approved by the ethics committee of the National Center for Geriatrics and Gerontology. Prior to participation, informed consent was obtained from all participants.

### 2.2. Subjective Cognitive Decline

We used the Cambridge Mental Disorders of the Elderly Examination (CAMDEX) questionnaire [13] and the Subjective Memory Complaints scale to assess SCD; they have also been used in previous studies [14]. A positive response to any of the following questions indicates SCD: (1) “Do you have any difficulty with your memory?”; (2) “Do you forget where you have left things more than you used to?”; (3) “Do you forget the names of close friends or relatives?”; and (4) “Do other people find you forgetful?”. For each question, participants answered yes = 1 or no = 0. A higher total score indicated greater SCD.

### 2.3. Frailty Assessment

Frailty was assessed using the Kihon Checklist (KCL), a Japanese frailty index, which constitutes a self-reported comprehensive health questionnaire. The KCL was developed to evaluate the risk of dependency among older adults. This questionnaire contains 25 yes/no questions divided into seven domains: lifestyle, physical strength, nutrition, eating, socialization/isolation, memory, and depressive mood. A total score of 4–7 is characteristic of a prefrail individual, whereas scores of 8 and above identify a frail individual [9]. The KCL was validated as a screening tool for frailty and has good to excellent accuracy: the area under the receiver operating characteristic curve to predict frailty (defined by the Fried criteria) was 0.92 in a sample of geriatric outpatients with chronic diseases and 0.88 in a sample of community-dwelling older adults [9].

### 2.4. Other Factors at Baseline

Information on medical history including heart disease, hypertension, diabetes, hyperlipidemia, and respiratory disease as well as age, sex, educational history, and whether they lived alone was obtained through a face-to-face interview. The body mass index was calculated by dividing their body weight (kg) by the square of their body height (m^2^). Gait speed was measured in m/s by asking the participants to navigate a straight, 6.4 m walking path at their usual gait speed. Gait time was measured in seconds over a 2.4 m distance between marks at 2.0 m and 4.4 m from the start of the walking path. Depressive symptoms were measured using the 15-item Geriatric Depression Scale (GDS) [15]. These factors have shown associations with frailty and SCD in previous studies [11,16,17,18,19].

### 2.5. Statistical Analyses

Group-based trajectory models were used to assess the trajectories of both frailty and SCD within the study sample over time. Group-based trajectory analysis, also known as latent class trajectory analysis or finite mixture modeling, is a statistical method used to identify and describe distinct subgroups or clusters within a larger population based on their trajectories over time. This method is commonly employed to analyze longitudinal data, where observations are collected from the same individuals or entities at multiple time points [20]. Here, change over time is considered for a heterogeneous mixture of groups, each with a distinct functional form (e.g., linear, quadratic). The models were developed using the procedure “traj” written for Stata [20]. We then fit a joint trajectory model, which provided probabilities regarding membership across the trajectory groups of frailty and SCD. This joint model helped determine a summary of the dynamic interrelationship between two longitudinal variables across various trajectory groups. To determine the number of groups, we initially used an intercept model for all groups. The final number of groups was determined based on the Bayesian information criterion (BIC), trajectory shapes for similarity, the proportion of membership in each group, and minimum posterior probabilities of group assignment (0.70) [16,21]. Finally, we identified three distinct frailty and SCD joint trajectories over a six-year period. To determine the number of frailty and SCD joint trajectory groups, although the BIC was slightly higher for the four and five group models than for the three group models, we chose three groups. This was because the trajectories between the groups had been covered, and four and five groups would involve splitting the largest group that did not show distinctive patterns. Furthermore, we determined the highest model functions of the three trajectory groups. The level of the polynomial for each group was reduced until a parameter estimated in the highest function had a *p*-value less than 0.01 [21]. The final model for frailty and SCD that met the selection criteria contained one constant trajectory, one quadratic trajectory, and one linear trajectory. Table 1 shows the model search process for frailty and the SCD joint trajectory model. Additionally, we adapted the dropout model. Intermittent missing data were treated as missing at random. The dropout model calculates trajectory-specific dropout probabilities based on previous wave observations and adjusts for group-specific membership probabilities. Group-based trajectory analyses were performed using Stata 14 mp (Stata Corp., College Station, TX, USA). Baseline characteristics were compared across groups and analyzed using one-way analysis of variance for continuous variables and chi-square tests for categorical variables. Multivariate logistic regression analysis was performed to examine the effect of related factors at baseline for each group of frailty and SCD. Furthermore, to examine the related factors of participants belonging to both frailty and SCD groups, we created a combined group and performed multivariate logistic regression to assess factors associated with membership in this combined group. The combined groups were as follows: (1) a group belonging to both non-progressive frailty and non-SCD, (2) a group belonging to both the moderate progressive frailty and the SCD group, and (3) a group belonging to both the rapid progressive frailty and SCD. Analyses were conducted using the IBM SPSS Statistics software package (25.0; SPSS Inc., Chicago, IL, USA). Statistical significance was set a priori at *p* < 0.05.

## 3. Results

**Figure 1:**
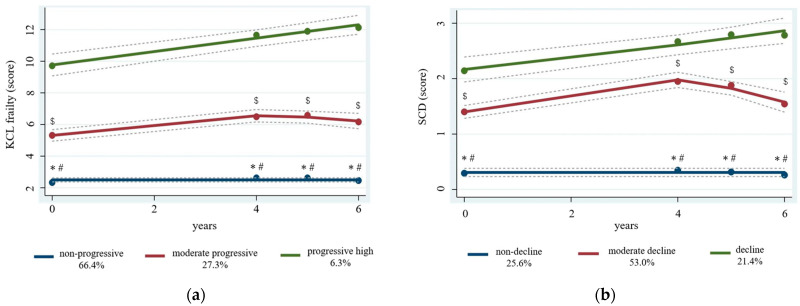
Mean KCL frailty scores and SCD scores and 95% confidence intervals. (**a**) Mean KCL frailty scores and 95% confidence intervals by KCL frailty. Dots indicate actual mean KCL frailty scores, the solid line indicates estimated mean KCL frailty scores, and the dotted line indicates 95% confidence intervals of estimated mean KCL frailty. KCL, Kihon Checklist. KCL frailty scores at each time point were compared between groups by one-way ANOVA. Significant difference was obtained by Bonferroni post hoc test. * *p*-values, non-progressive frailty vs. moderate progressive frailty; ^#^ *p*-values, non-progressive frailty vs. progressive high frailty; ^$^ *p*-values, moderate progressive frailty vs. progressive high frailty. (**b**) Mean SCD scores and 95% confidence intervals by SCD. Dots indicate actual mean SCD scores, the solid line indicates estimated mean SCD scores, and the dotted line indicates 95% confidence intervals of estimated mean SCD. SCD, subjective cognitive decline. SCD scores at each time point were compared between groups by one-way ANOVA. Significant difference was obtained by Bonferroni post hoc test. * *p*-values, non-decline SCD vs. moderate decline SCD; ^#^ *p*-values, non-decline SCD vs. decline SCD; ^$^ *p*-values, moderate decline SCD vs. decline SCD.

We labeled the frailty trajectory groups as follows: group 1 = non-progressive frailty, group 2 = moderate progressive frailty, and group 3 = rapid progressive frailty; and the SCD trajectory groups as follows: group 1 = non-progressive SCD, group 2 = moderate progressive SCD, and group 3 = rapid progressive SCD. Figure 1a describes the three frailty trajectory groups over time: non-progressive frailty (66.4%), moderate progressive frailty (27.3%), and rapid progressive frailty (6.3%). Posterior probabilities of group membership were 0.94 (non-progressive frailty), 0.86 (moderate progressive frailty), and 0.91 (rapid progressive frailty). Furthermore, KCL frailty scores and SCD scores at each time point were compared between groups by one-way ANOVA. There was a significant difference between the three groups in KCL frailty scores (all < 0.001). Regarding SCD scores, a significant difference was found between the three groups at all time points (all < 0.001). Figure 1b shows the SCD trajectory groups: non-progressive SCD (25.6%), moderate progressive SCD (53.0%), and rapid progressive SCD (21.4%). Posterior probabilities of group membership were 0.82 (non-progressive SCD), 0.83 (moderate progressive SCD), and 0.81 (rapid progressive SCD).

Table 2 shows the demographics and health characteristics of the study participants at baseline. At baseline, the non-progressive frailty group was younger (*p* < 0.001), comprised fewer women (*p* < 0.001), had lower GDS scores (*p* < 0.001), had faster gait speed (*p* < 0.001), and had a lower frequency of hypertension (*p* = 0.013). Conversely, the rapid progressive frailty group had a higher frequency of heart disease (*p* < 0.001), hyperlipidemia (*p* = 0.021), and respiratory disease (*p* = 0.040) and was living alone (*p* = 0.003). Regarding SCD, the rapid progressive SCD group was likely to have heart disease (*p* = 0.029), hyperlipidemia (*p* = 0.022), higher GDS scores (*p* < 0.001), and lower gait speed (*p* = 0.012). 

The group most likely to be in non-progressive frailty was non-progressive SCD (86.1%). Furthermore, belonging to rapid progressive SCD was associated with a 32.2% chance of belonging to rapid progressive frailty. However, non-progressive SCD, on the other hand, had no chance of belonging to rapid progressive frailty (Table 3). 

The results of the multinomial logistic regression predicting the odds ratio of trajectory groups for both frailty and SCD are shown in Table 4. Women, individuals with high GDS scores, and having faster gait speed were associated with lower odds of both the moderate progressive and rapid progressive frailty groups than the non-progressive frailty group in the frailty models. When compared with the non-progressive frailty group, having heart and respiratory diseases was associated with an increased likelihood of membership in rapid progressive frailty groups. Individuals with higher levels of education and lower GDS scores had higher odds of moderate and rapidly progressive SCD groups in the SCD trajectory model than in the non-progressive SCD group. Women were more likely to be in the rapid progressive SCD group than the non-progressive SCD group. Furthermore, faster gait speed was related to a lower risk of belonging to the rapid progressive SCD group vs. the non-progressive SCD group. 

Table 5 shows the results of the predicted odds for belonging to both the moderate progressive frailty and SCD groups, and both the rapid progressive frailty and SCD groups, compared with both the non-progress frailty and SCD groups. Women and people with high GDS scores were more likely to be in the moderate and rapid progressive frailty and SCD groups than in the non-progressive frailty and SCD groups. When compared with non-progressive frailty and SCD, faster gait speed was related to a lower risk of both the moderate and rapid progressive frailty and SCD groups.

## 4. Discussion

We identified three distinct KCL frailty scores and SCD score trajectory patterns (non, moderate, and rapid) among Japanese older adults over 70 years old. The non-progressive frailty group or the non-SCD group had lower KCL frailty scores or SCD scores at baseline. The moderate progressive frailty group or the moderate SCD group had intermediate KCL frailty scores or SCD scores at baseline. Individuals in the rapid progressive frailty group or the rapid SCD group had the highest KCL frailty scores or SCD scores at baseline. These findings suggest that older adults’ physical or cognitive status at baseline may determine their health in the future. Therefore, implementing strategies to improve both physical and cognitive functioning for those with moderate to severe decline is crucial during screening.

Our results showed that membership in the non-SCD group was associated with a high probability of being a member in the non-progressive frailty group (86.1%). Moreover, membership in the rapid SCD group was associated with a 32.2% probability of being in the rapid progressive frailty group, whereas membership in the non-SCD group had zero probability of belonging to the rapid progressive frailty group. This suggests an overlap between frailty and cognitive decline, which supports previous studies that link frailty and cognitive decline [22]. Frailty and MCI are usually considered separate concepts; however, they tend to be comorbid in later life, interacting with each other and having a cumulatively adverse effect on health, resulting in significant adverse outcomes [22]. Moreover, the results of the prospective cohort study indicate that individuals with mild cognitive impairment at baseline have a higher likelihood of developing frailty, affecting the trajectory of frailty, and vice versa [23,24]. Recent systematic review and meta-analyses showed that the components of frailty, comprising exhaustion, weight loss, slowness, weakness, and low physical activity, overlap in part with the definition of reduced quality of life and have a serious impact on the physical function, energy, social functioning, and mental health of the elderly, which is further deteriorated when combined with mild cognitive impairment. This review suggests that it is important to have more effective strategies for the prevention and management of frailty and MCI in an aging society [25]. Frailty and cognitive decline co-occur and interact mutually in later life; the prevalence of the co-occurrence of frailty and cognitive decline is associated with adverse health outcomes [12,26]. Xue et al. (2021) found that cognitive function not only directly influences health outcomes but also indirectly influences health outcomes through frailty [27]. Possible explanations for this co-occurrence include AD-related plaque development, cardiovascular disease, nutritional imbalance, and chronic inflammatory disease. It is plausible that cognitive decline and frailty share a common underlying pathology [8]. Our results, combining trajectories of rapidly increasing frailty with rapid cognitive decline, further support the hypothesis of a shared underlying pathology.

Our findings showed that those in the combined rapid progressive frailty and SCD groups were associated with a higher risk of depressive symptoms. Additionally, the groups with more progressive conditions in terms of frailty or SCD were associated with a gradually increased risk in depressive symptoms. Studies show that there is a relationship between depression and cognitive impairment alone [24]. Recent large-scale study by the Wu et al. (2022) suggests that chronic conditions such as depression and behavioral factors may be helpful in maintaining cognitive function in the elderly [28]. SCD has been found to be consistent with the preclinical phase of the AD framework, indicating the critical period between the stage of no cognitive impairment and the stage of cognitive impairment [3]. Memory complaints are often reported in individuals with depressive symptoms, and depressive symptoms are frequent in the early stages of AD [18]. A systematic review suggests that a high proportion of older adults who are frail have depressive symptoms [19]. Frailty, cognitive dysfunction, and depression are interrelated components [29] and share several underlying pathophysiologic mechanisms, including chronic inflammation, subclinical cerebrovascular diseases, and hypothalamic pituitary axis stress response dysfunction [19]. Our results suggest that clinicians should provide holistic assessment and care in terms of the physical, cognitive, and psychological aspects to meet the multidimensional healthcare needs of older adults. Furthermore, it suggests the importance of screening depressive symptoms and providing strategies for promoting mental health in later life. 

Additionally, we found that low gait speed was associated with progressive frailty and rapid SCD. Reduced usual walking speed is a predictor of frailty [17]. Recent study involving more than 19,000 community-dwelling older adults observed that gait speed was positively related to the likelihood of high cognitive trajectories and negatively with the risk of low cognitive trajectories. This result suggests that gait speed was a stringer predictor of cognitive decline trajectory in men [30]. The walking ability is a valuable predictor of functional decline in older adults. Our results suggest that gait speed can be considered a common variable for assessing functional ability in older adults. Although many prospective studies in the general population show that slow gait speed is associated with objective cognitive decline and an increased risk in incident dementia, studies on gait speed in relation to SCD are scarce [31]. SCD is the earliest clinically detectable stage of cognitive change that may lead to dementia. A previous cross-sectional study found that the usual walking speed was significantly slower in older adults with SCD than in controls [32]. Thus, reduced walking speed in individuals with SCD may be related to complex motor tasks and reduced cognitive abilities in the early stages of neurodegeneration [32]. This suggests that slow gait speed may be an early marker of cognitive decline. 

Similar to a previous study, our results showed that combined rapid progressive frailty and SCD was primarily seen in women as compared with men [33]. The mechanisms underlying the development of frailty and cognitive changes, such as oxidative stress, inflammation, and hormone levels (estrogen and testosterone), have been proposed to differently affect interrelated organ systems in women and men [34,35]. Furthermore, we found that participants who complained about their subjective cognition had higher education levels, suggesting that highly educated individuals might notice subtle changes in their performance. 

A major strength of this study is that it examined the joint trajectories of frailty and SCD based on longitudinal data in community-dwelling older adults. Our study has several limitations. First, the baseline and consequent assessment were administered four years apart. In this time, unmeasured increased frailty and cognitive decline may have occurred. Thus, our model may have underestimated the true rate of the decline. Second, participants were from a representative sample of Japanese older adults, limiting the generalizability of our results. Third, we included only survivors, which may have influenced the estimates of frailty and SCD. This study followed a six-year observation period, which may not be enough to detect apparent frailty; thus, any suggested association must be interpreted with caution. Fourth, self-report questionnaires were used to evaluate frailty and SCD. Future studies can employ objective measures for evaluating frailty or cognitive decline and compare their results with this study.

## 5. Conclusions

Our results have found that frailty and SCD share a similar trajectory in Japanese older adults; in other words, the rate of decline for both conditions seemed to be similar. Members of the rapid progressive frailty group had the highest probability of membership in the rapid SCD group, suggesting that frailty is accompanied with cognitive decline. Additionally, rapid progressive frailty and SCD were associated with the highest risk of depressive symptoms and slow gait speed. Thus, interventions to reduce rapid decline in both frailty and cognition might benefit from focusing on mental health and increasing gait speed. These findings will aid the design and selection of frailty and dementia prevention and intervention programs by Japanese healthcare professionals in the geriatrics field.

## Figures and Tables

**Table 1:** Model search process for frailty and SCD trajectories.

Determining the number of frailty and SCD trajectories												
Number of groups		BIC		Smallest group %								
				Frailty		SCD						
1		−16,063		100.0		100.0						
2		−15,186		13.7		35.5						
3		−15,003		4.8		19.0						
4		−14,886		3.1		13.1						
5		−14,799		2.9		10.8						
Three frailty and SCD trajectory groups were chosen.												
Determining the highest model function of the three frailty and SCD trajectory groups.												
	Frailty						SCD					
Group	1st iteration		2nd iteration		3rd iteration		1st iteration		2nd iteration		3rd iteration	
	Highest function	*p*-value	Highest function	*p*-value	Highest function	*p*-value	Highest function	*p*-value	Highest function	*p*-value	Highest function	*p*-value
Group 1	Quadratic	0.050	Linear	0.020	Constant	<0.001	Quadratic	0.044	Linear	0.862	Constant	<0.001
Group 2	Quadratic	<0.001	Quadratic	<0.001	Quadratic	<0.001	Quadratic	<0.001	Quadratic	<0.001	Quadratic	<0.001
Group 3	Quadratic	0.292	Linear	<0.001	Linear	<0.001	Quadratic	0.328	Linear	<0.001	Linear	<0.001

Note: BIC, Bayesian information criterion; SCD, subjective cognitive decline.

**Table 2:** Demographics of the study participants at baseline according to the trajectories of frailty and SCD.

	Frailty Groups					SCD groups				
	Non-Progressive Frailty	Moderate Progressive Frailty	Rapid Progressive Frailty	*p*-Value	Post Hoc	Non-Progressive SCD	Moderate Progressive SCD	Rapid Progressive SCD	*p*-Value	Post Hoc
*n*	775	312	70			302	625	230		
Age, years	74.9 ± 3.7	75.9 ± 4.1	76.0 ± 4.3	<0.001 *	Non < Moderate, Rapid	74.9 ± 3.9	75.4 ± 3.9	75.2 ± 3.7	0.149	
Female, *n* (%)	343 (44.3) ^§^	178 (57.1) ^‡^	41 (58.6)	<0.001 ^†^		136 (45)	302 (48.3)	124 (53.9)	0.125	
BMI, kg/m^2^	23.0 ± 2.8	23.1 ± 3.2	22.8 ± 3.5	0.648		23.3 ± 3.0	23.0 ± 2.9	22.8 ± 2.7	0.138	
Education, years	13.1 ± 2.6	12.5 ± 2.4 ^‡^	12.8 ± 2.8	0.002 *	Non > Moderate	12.5 ± 2.4	13.1 ± 2.7	12.9 ± 2.6	0.004 *	Non < Moderate
Living alone, *n* = yes (%)	103 (13.3) ^§^	56 (17.9)	19 (27.1) ^‡^	0.003 ^†^		44 (14.6)	92 (14.7)	42 (18.3)	0.401	
Heart disease, *n* = yes (%)	136 (17.5) ^§^	74 (23.7)	24 (34.3) ^‡^	<0.001 ^†^		56 (18.5)	117 (18.7)	61 (26.5) ^‡^	0.029 ^†^	
Hypertension, *n* = yes (%)	335 (43.2) ^§^	162 (51.9) ^‡^	38 (54.3)	0.013 ^†^		131 (43.4)	293 (46.9)	111 (48.3)	0.478	
Diabetes disease, *n* = yes (%)	80 (10.3)	45 (14.4)	9 (12.9)	0.152		30 (9.9)	72 (11.5)	32 (13.9)	0.363	
Hyperlipidemia, *n* = yes (%)	303 (39.1)	133 (42.6)	39 (55.7) ^‡^	0.021 ^†^		110 (36.4)	254 (40.6)	111 (48.3) ^‡^	0.022 ^†^	
Respiratory disease, *n* = yes (%)	138 (17.8)	63 (20.2)	21 (30.0) ^‡^	0.040 ^†^		51 (16.9)	118 (18.9)	53 (23)	0.195	
GDS, score	1.7 ± 1.8	3.5 ± 2.7	6.7 ± 3.4	<0.001 *	Non < Moderate < rapid	1.5 ± 1.9	2.3 ± 2.3	4.3 ± 3.1	<0.001 *	Non < Moderate < Rapid
Gait speed, m/s	1.17 ± 0.20	1.09 ± 0.20	1.06 ± 0.23	<0.001 *	Non > Moderate, Rapid	1.16 ± 0.19	1.14 ± 0.21	1.10 ± 0.20	0.012 *	Non, Moderate > Rapid

Note: SCD, subjective cognitive decline; BMI, body mass index; GDS, Geriatric Depression Scale. * *p*-values reported by one-way ANOVA. Significant difference was obtained by Bonferroni post-hoc test. ^†^ *p*-values obtained by Pearson’s chi-square test. ^‡^ Statistically significant association by adjusted standardized residual > 1.96 (*p* < 0.05). ^§^ Statistically significant association by adjusted standardized residual < −1.96 (*p* < 0.05).

**Table 3:** Probability of membership in an SCD trajectory group given membership in a frailty trajectory group.

	Frailty Groups		
SCD Groups	Non-Progressive Frailty	Moderate Progressive Frailty	Rapid Progressive Frailty
Non-progressive SCD	86.1%	13.9%	0.0%
Moderate progressive SCD	62.5%	37.5%	0.0%
Rapid progressive SCD	4.0%	63.8%	32.2%

Note: SCD, subjective cognitive decline.

**Table 4:** Logistic regression predicting odds of belonging to frailty or SCD.

	Frailty Groups					SCD Groups				
	Non-Progressive Frailty	Moderate Progressive Frailty		Rapid Progressive Frailty		Non-Progressive SCD	Moderate Progressive SCD		Rapid Progressive SCD	
*n*	775	312		70		302	625		230	
Group Probability	0.94	0.86		0.91		0.82	0.83		0.81	
	Ref	OR (95% CI)	*p*-Value	OR (95% CI)	*p*-Value	Ref	OR (95% CI)	*p*-Value	OR (95% CI)	*p*-Value
Age, years		1.04 (1.04 to 1.08)	0.039	1.03 (0.95 to 1.11)	0.507		1.04 (1.00 to 1.08)	0.078	0.99 (0.94 to 1.05)	0.821
Female, *n* (%)		2.04 (1.46 to 2.83)	<0.001	2.53 (1.28 to 5.00)	0.008		1.35 (0.98 to 1.86)	0.068	1.71 (1.11 to 2.62)	0.014
BMI, kg/m^2^		1.01 (0.96 to 1.07)	0.657	0.97 (0.87 to 1.07)	0.531		0.97 (0.92 to 1.02)	0.186	0.94 (0.88 to 1.00)	0.063
Education, years		0.98 (0.92 to 1.04)	0.479	1.09 (0.97 to 1.23)	0.166		1.14 (1.08 to 1.21)	<0.001	1.17 (1.08 to 1.27)	<0.001
Living alone, yes		0.95 (0.63 to 1.44)	0.805	1.7 (0.84 to 3.44)	0.144		0.84 (0.55 to 1.29)	0.424	0.94 (0.55 to 1.61)	0.831
Heart disease, yes		1.41 (0.98 to 2.02)	0.063	2.55 (1.32 to 4.94)	0.005		0.94 (0.65 to 1.37)	0.758	1.4 (0.88 to 2.23)	0.156
Hypertension, yes		1.26 (0.92 to 1.72)	0.15	1.08 (0.57 to 2.05)	0.804		1.17 (0.86 to 1.58)	0.317	1.08 (0.72 to 1.61)	0.73
Diabetes disease, yes		1.34 (0.86 to 2.09)	0.191	1.27 (0.54 to 2.95)	0.587		1.17 (0.73 to 1.88)	0.503	1.38 (0.77 to 2.47)	0.284
Hyperlipidemia, yes		0.81 (0.60 to 1.10)	0.183	0.99 (0.54 to 1.82)	0.97		1.07 (0.79 to 1.44)	0.668	1.13 (0.76 to 1.68)	0.532
Respiratory disease, yes		1.11 (0.77 to 1.61)	0.566	2.04 (1.04 to 4.00)	0.039		1.03 (0.71 to 1.50)	0.884	1.31 (0.82 to 2.10)	0.266
GDS, score		1.42 (1.33 to 1.52)	<0.001	1.94 (1.75 to 2.16)	<0.001		1.23 (1.13 to 1.33)	<0.001	1.58 (1.44 to 1.73)	<0.001
Gait speed, m/s		0.27 (0.12 to 0.58)	0.001	0.1 (0.02 to 0.49)	0.004		0.87 (0.41 to 1.84)	0.716	0.36 (0.13 to 0.97)	0.043

Note: SCD, subjective cognitive decline; OR, odds ratio; CI, confidence interval; Ref, reference group; BMI, body mass index; GDS, 15-item geriatric depression scale.

**Table 5:** Logistic regression predicting odds of belonging to the moderate progressive frailty and moderate SCD group, and the rapid progressive frailty and SCD group, compared to the non-progressive frailty and SCD group combinations.

	Non-Progress Frailty and SCD	Moderate Progressive Frailty and SCD		Rapid Progressive Frailty and SCD	
*n*	290	141		53	
	Ref	OR (95% CI)	*p*-Value	OR (95% CI)	*p*-Value
Age, years		1.07 (1.01 to 1.14)	0.018	0.98 (0.88 to 1.09)	0.686
Female, *n* (%)		2.72 (1.60 to 4.65)	<0.001	2.59 (1.04 to 6.46)	0.040
BMI, kg/m^2^		1.01 (0.94 to 1.08)	0.860	0.94 (0.83 to 1.07)	0.348
Education, years		1.08 (0.96 to 1.19)	0.143	1.35 (1.14 to 1.59)	0.001
Living alone, yes		0.80 (0.43 to 1.51)	0.490	1.95 (0.76 to 5.06)	0.167
Heart disease, yes		1.39 (0.79 to 2.45)	0.248	2.40 (0.99 to 5.84)	0.053
Hypertension, yes		1.27 (0.77 to 2.10)	0.349	0.59 (0.24 to 1.42)	0.240
Diabetes disease, yes		1.18 (0.57 to 2.41)	0.659	0.82 (0.26 to 2.57)	0.739
Hyperlipidemia, yes		0.90 (0.55 to 1.47)	0.681	2.18 (0.97 to 4.93)	0.060
Respiratory disease, yes		0.99 (0.55 to 1.77)	0.960	2.04 (0.85 to 4.91)	0.111
GDS, score		1.48 (1.32 to 1.66)	<0.001	2.14 (1.82 to 2.53)	<0.001
Gait speed, m/s		0.15 (0.04 to 0.50)	0.002	0.04 (0.00 to 0.32)	0.003

Note: SCD, subjective cognitive decline; OR, odds ratio; CI, confidence interval; Ref, reference group; BMI, body mass index; GDS, 15-item Geriatric Depression Scale.

## Data Availability

Qualified researchers can obtain the data from the corresponding author (srbae@dau.ac.kr). The data are not publicly available due to privacy concerns imposed by the IRB.
